# Visual and optical quality outcomes of SMILE and FS-LASIK for myopia in the very early phase after surgery

**DOI:** 10.1186/s12886-019-1096-z

**Published:** 2019-04-08

**Authors:** Ting Liu, Guanting Lu, Kaijian Chen, Qiuxia Kan, Ji Bai

**Affiliations:** 1 Department of Ophthalmology, Daping Hospital of Army Medical University of PLA, No. 10 Changjiangzhi Road, Yuzhong District, Chongqing, 400042 China; 2 Department of Blood Transfusion, Tangdu Hospital, Fourth Military Medical University, Xi’an, China

**Keywords:** Myopia, SMILE, FS-LASIK, very early phase

## Abstract

**Background:**

Small incision lenticule extraction (SMILE) and femtosecond laser-assisted in situ keratomileusis (FS-LASIK) are frequently used to treat myopia. However, little is known about the impact on recovery of these approaches in the very early postsurgical phase (within 24 h).

**Methods:**

To compare the efficacy of these two procedures for the treatment of myopia in the early phase after surgery, differences in visual acuity, OSI (objective scattering index), cutoff for modulation transfer function (MTF), and SR (Strehl ratio) between SMILE and FS-LASIK were evaluated at 0, 2, 4 and 24 h postoperatively using two-way analysis of variance (ANOVA).

**Results:**

No significant differences between SMILE and FS-LASIK in the MTF cutoff and SR were found (*p* > 0.05). However, at 2 h and 4 h after surgery, OSI values in the SMILE group were significantly higher than those in the FS-LASIK group, and visual acuity scores in the SMILE group were significantly poorer than those in the FS-LASIK group (*p* < 0.05). Regarding subjective symptoms, the number of patients complaining of eye dryness, blurred vision, foreign body sensation and eye soreness in the SMILE group were lower than the number in the FS-LASIK group.

**Conclusions:**

In conclusion, visual and optical quality outcomes of FS-LASIK for myopia were better than those of SMILE in the very early phase after surgery, a difference that is attributable to the formation of interface haze.

**Trial registration:**

ChiCTR1900021451.

## Background

Currently, small incision lenticule extraction (SMILE) procedure and femtosecond laser-assisted in situ keratomileusis (FS-LASIK) are two popular techniques used in the clinical treatment of myopia and myopic astigmatism [1–4]. Used to treat refractive errors for more than a decade, FS-LASIK possesses many advantages over mechanical microkeratomes for such treatment. First, surgeons can control the flap diameter, thickness and hinge position and width [5]. Second, FS-LASIK can also reduce the occurrence of certain complications, such as buttonholes as well as irregular and partial flaps [6]. However, FS-LASIK can also lead to complications, such as dry eye syndrome [7, 8].

SMILE, a new type of procedure for treating refractive errors, was developed on the basis of FS-LASIK. Because FS-LASIK appears to be associated with the risk of postoperative corneal flap complications, SMILE is regarded as a safer procedure by some ophthalmologists [9]. Nonetheless, compared with LASIK, it has been postulated that a longer period of time is required for visual recovery when using SMILE. Although both SMILE and FS-LASIK are safe and effective, with predictable clinical parameters during months of follow-up [9], little is known about the clinical parameters in the very early stage of recovery (within 24 h) in patients who have undergone these procedures.

Optical Quality Analysis System II (OQAS, Visiometrics, Terrassa, Spain) is used to collect retinal images derived from an electric light source and to analyze their point spread function (PSF) characteristics using the dual-channel technique. A series of visual quality-related indexes such as the Objective Scattering Index (OSI), Cutoff for Modulation Transfer Function (MTF), and Strehl ratio (SR) is produced, and compared with vision or contrast sensitivity, these measures can better reflect the muddy degree of the refractive media of human eyes and the source of visual quality problems objectively.

To compare the efficacy of the two above procedures for the treatment of myopia in the early phase after surgery, we conducted this prospective study in which we evaluated postoperative visual recovery after SMILE and LASIK.

## Methods

### Patients

This retrospective clinical study received approval from the Ethics Committee of Daping Hospital and the Research Institute of Surgery of the Third Military Medical University. Written informed consent was obtained from all of the participants prior to the time of intervention.

Thirty subjects (60 eyes) at Chongqing Daping Hospital between January to August 2016 were enrolled in each group (SMILE and FS-LASIK). All of the subjects (both eyes) voluntarily underwent SMILE or LASIK. The inclusion criteria were: 1) corrected distance visual acuity (CDVA) 20/20 (Snellen); 2) sufficient corneal thickness; 3) age > 18 years and < 35 years; 4) the change in diopter was < 0.5 D within 2 years before the operation;and 5) without a history of ocular surgery, severe dry eye, progressive corneal degeneration, cataract, or uveitis. There were no significant differences between the two groups in CDVA, spherical equivalent (SE), central corneal thickness (CCT) and intraocular pressure (IOP). Age and gender distribution were matched in each group, with no significant differences.

### Procedures

All patients received preoperative topical antibiotic eye drops (0.3% tobramycin eye drops, twice daily for 3 days). Preoperative surface anesthesia was administered (0.4% oxybuprocaine hydrochloride eye drops) (Benoxil; Santen Pharmaceuticals, Japan).

SMILE procedures were performed using the VisuMax femtosecond laser (Zeiss, Oberkochen, Germany). The following femtosecond laser parameters were used: 120 μm cap thickness, 7.5 mm diameter of the cap, and 6.5 mm diameter of the posterior lenticule surface. A 2-mm-long (32°) corneal incision was made at 12 o’clock position. The remaining tissue bridges were broken using a thin, blunt spatula, and the lenticule was then grasped and removed through the small incision with a microforceps.

The WaveLight suite, FS200 femtosecond and EX500 excimer laser (Alcon Laboratories, Inc., Fort Worth, TX) was used for all FS-LASIK procedures. For all cases, the flap diameter was 8.0 mm, and the flap thickness was 110 μm. The track and spot distances were 3.0 μm during flap creation and 1.5 μm during flap side-cutting. Stromal ablation was performed with EX500 excimer laser using a 6.5 mm optical zone. The flap was repositioned and the stromal bed was washed with balanced salt solution after laser ablation of the stromal bed. All SMILE and LASIK procedures were performed by the same experienced surgeon (JB).

Levofloxacin Eye Drops (Santen, Japan) and Tobramycin Dexamethasone Eye Drops (Alcon Laboratories, Inc., Fort Worth, TX) were applied immediately after procedures, and no other interventions were performed within 24 h. Anti-infection (Levofloxacin Eye Drops, Santen Japan, three times a day, 3–4 weeks)anti-inflammatory treatment (0.5% loteprednol etabonate ophthalmic suspension, Bausch & Lomb USA; four times daily for 1 week, followed by twice daily for 3–4 weeks), and sodium hyaluronate eye drops (URSAPHARM Arzneimittel GmbH, Germany; four times a day, start at week two and lasted for 3 months) were applied.

### Measurement of visual indicators

CDVA [10] was routinely measured at every visit, and time-course changes in CDVA during the ocular convalescent stage from the onset of disease were obtained from clinical records. CDVA was determined using a standard Landolt visual acuity (VA) chart and then converted to a logarithm of the minimal angle resolution (LogMAR) visual acuity for statistical analyses. The measurement was conducted four times: 24 h before surgery, 2 h after surgery, 4 h after surgery, and 24 h after surgery.

### Statistical analysis

All statistical analyses were performed using SPSS 18.0 (SPSS Inc., Chicago, Illinois, USA). The independent samples t-test was applied to compare differences in OSI, MTF cutoff, and SR between the SMILE and LASIK groups at different time points. The ratio of visual acuity of < 0.3 (LogMAR 0.525) and < 0.1 (LogMAR 1.0) at different time points and the ratio of patients with subjective symptoms (e.g., eye dryness, blurred vision, foreign body sensation, eye soreness) in the groups were calculated. Unless otherwise indicated, a value of *p* < 0.05 was considered statistically significant.

## Results

### Study subjects

Each of the 30 subjects underwent LASIK or SMILE. The demographics of the study population are summarized in Table 1. No significant differences were detected in terms of CDVA, SE, or IOP. The distributions of age and sex for the two groups were very similar, with no significant differences. All of the operations were successful, with no intraoperative complications.

**Table 1:** Demographic information of the two groups before surgery

	LogMAR	SE (D)	IOP (mmHg)	Age	Sex	
					Female	Male
FS-LASIK	−0.01 ± 0.05	−5.70 ± 1.83	13.30 ± 1.71	22.43 ± 4.02	17	13
SMILE	0.00 ± 0.07	−5.40 ± 2.21	13.12 ± 1.56	24.56 ± 5.32	20	10
*T* value	0.42	− 0.6	1.78	−1.27		
*p* value	> 0.05	> 0.05	> 0.05	> 0.05		

### Efficacy of treatment

**Fig. 1:**
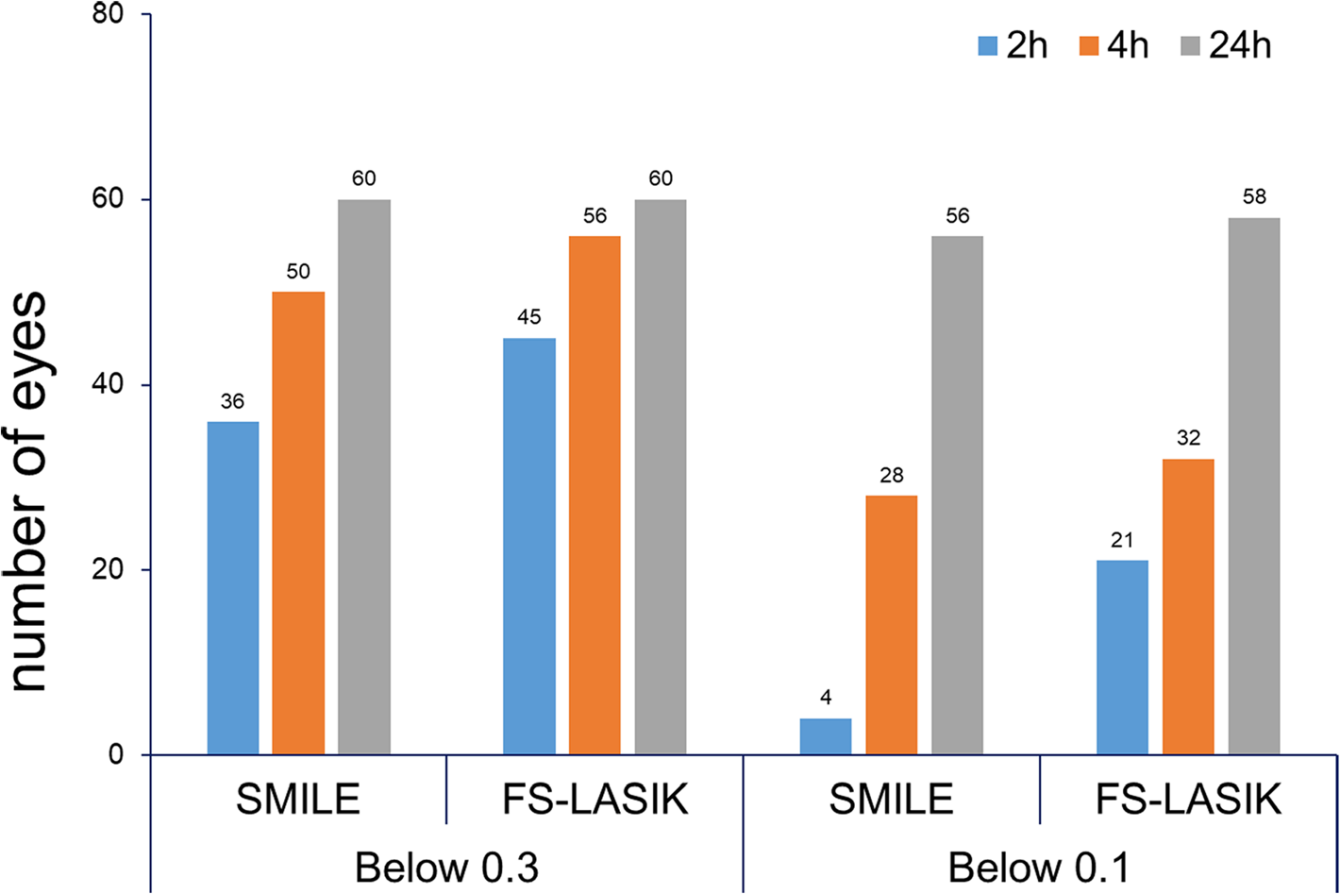
Number of eyes recovering below 0.3 (LogMAR 0.525) and 0.1 (LogMAR 1.0) among patients undergoing SMILE or FS-LASIK

To study the efficacy of SMILE and FS-LASIK, we conducted a visual test for each group. As shown in Fig. 1, the visual acuity of all of the patients had recovered to below 0.3 at 24 h after operation, indicating that both procedures were effective in the treatment of myopia. However, it should be noted that at 2 h and 4 h after surgery, the vision of patients who underwent SMILE was generally poor compared with those who underwent FS-LASIK. In the below 0.3 group, the visual acuity of 36 eyes (60%) treated with SMILE reached this level at 2 h after surgery, which was relatively lower than that of the eyes subjected to the LASIK procedure (75%). At 4 h postsurgery, the eye sight of 83.3% of the eyes treated by SMILE was below 0.3, whereas more than 93% of the eyes treated with LASIK was below 0.3; after 24 h, the visual acuity of all of the subjects was less than 0.3. For the 0.1 group, only 4 eyes (6.7%) subjected to SMILE reached this level at 2 h postsurgery, which was only approximately 19% of the eyes treated with LASIK (35%). However, at 4 and 24 h after surgery, the numbers of eyes reaching below 0.1 were increased more than 7 and 9 times compared with the numbers at the 2-h time point. Although the number of eyes that reached below 0.1 was greater among those subjected to LASIK, the increase was not as sharp as for those treated with SMILE.

### Comparison of clinical parameters

**Fig. 2:**
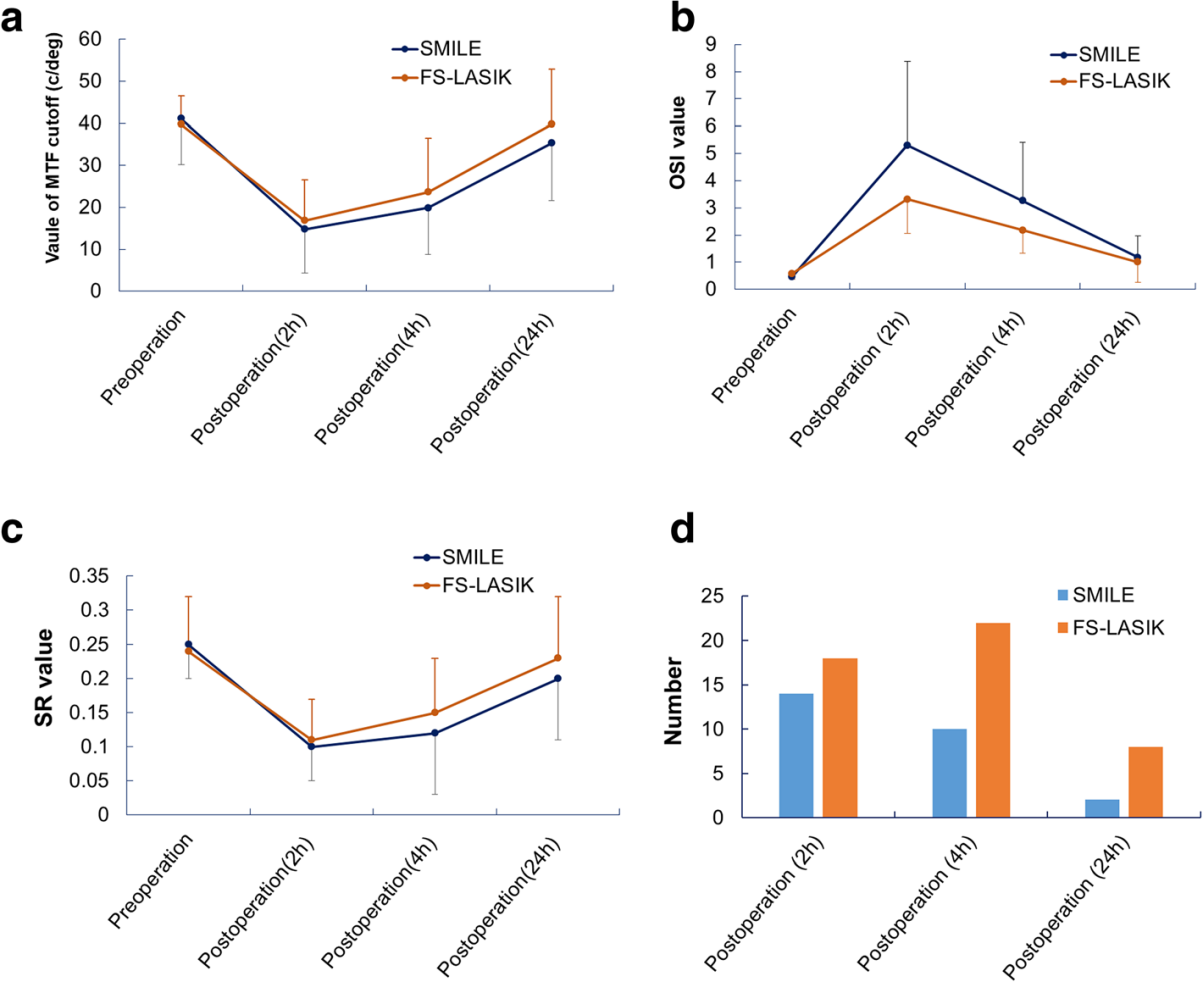
Comparison of clinical parameters for SMILE and FS-LASIK. **a**: comparison of MTF cutoff between the two groups. **b**: comparison of OSI between the two groups. **c**: comparison of SR between the two groups. **d**: comparison of incidence of subjective symptoms between the two groups

To obtain a panoramic view of the postoperative visual recovery process in the very early phase after treatment with SMILE and LASIK, we compared the MTF cutoff, OSI, and SR between the two groups. No significant differences in MTF cutoff (Fig. 2a) and SR (Fig. 2c) were observed before or after the operation at different time points, though OSI was significantly higher at 2 and 4 h after SMILE compared with after LASIK (Fig. 2b). We also investigated the incidence of subjective symptoms among the two groups. As shown in Fig. 2d, the incidence of subjective symptoms (including eye dryness, blurred vision, foreign body sensation and eye soreness) in patients who underwent SMILE decreased over time after surgery. In contrast, the incidence in patients who underwent FS-LASIK slowly increased at 4 h after surgery but decreased at 24 h after surgery. Overall, the incidence of subjective symptoms was higher for patients who underwent FS-LASIK than for those who underwent SMILE.

## Discussion

In this study, we compared time-dependent changes in clinical parameters between SMILE and FS-LASIK in the very early phase after the surgery. Considering their wide application for the treatment of myopia, it is of clinical importance to compare recovery indexes for these surgical procedures in this very early postoperative period. Although the sample size was small and the follow-up time was no more than 24 h, this study provides valuable information for comparing clinical outcomes of these surgical techniques, and to our knowledge, this is the first such report. For all of the patients included, recovery was satisfactory at 24 h after surgery. However, the vision of patients in the SMILE group was poor during the first few hours after surgery compared with that of patients in the FS-LASIK group.

SR indicates the ratio of the light intensity of the Gaussian image point between defective and perfect refractive media, which is 0.15 in healthy adults; the higher the value, the better is the visual quality. OSI is the ratio of light energy in peripheral images at 12–20 arc sec, which occurs in central images at 1 arc sec in the OQAS dual-channel imaging; the higher the value, the muddier the refractive media becomes. OSI is < 2 in adults with abnormal vision and is 2–4 in early cataract patients. The MTF cutoff means that in the MTF curves of human eyes, the resolving capacity would reach its peak when the spatial frequency is near this value. The MTF cutoff is ≥30 cpd in adults with normal vision; the higher the value, the better is the visual quality becomes.

In the current study, the vision of patients was poor, and OSI was larger in the SMILE group during the first few hours after surgery compared with that in the FS-LASIK group. However, the MTF cutoff and SR were not different in these two groups, indicating that during the first few hours after surgery, the difference in visual acuity may be caused by the muddiness of the refractive media, and this muddiness might be associated with the formation of interface haze.

Dry eye is one of the most common complications of LASIK surgery. The clinical signs of post-LASIK dry eye include positive vital staining of the ocular surface, decreased tear film breakup time and Schirmer’s test scores, reduced corneal sensitivity, and decreased functional visual acuity; these symptoms and signs can last at least 1 month after LASIK [11]. Indeed, it had been shown that approximately 19% of patients report dissatisfaction after being treated with FS-LASIK [12–14]. In one literature review, more than 50% of patients suffered from symptoms of dryness [8], but this percentage is likely high, as patients who suffered from a preoperative dry eye condition were included.

Preoperative eye dryness is a major risk factor for more severe dry eye after surgery and should be identified prior to any procedure. Previous studies report that thin-flap LASIK is associated with transient postoperative dry eye symptoms [15, 16]. Because the SMILE procedure only involves a small incision in the cornea, it is unlikely that the corneal nerves would be impaired. In FS-LASIK, a complete lamellar flap (only with a small hinge without cut) is created, and great disruption of the dense subbasal nerve plexus and stromal corneal nerves is caused, whereas in SMILE, there is less damage to the corneal nerves. This reduced damage might be the major reason for the lower reporting of dry eyes postoperatively. Regardless, a prospective, randomized clinical trial (contralateral-eye study) assessing corneal sensation measured by Cochet-Bonnet esthesiometry reported a significant decrease after both types of surgery, though with more pronounced effects after F-LASIK compared to SMILE. In one study comparing dry eye parameters such as tear breakup time, Schirmer’s test, and tear film osmolarity, no differences in dry eye symptoms were found between the two groups [17]. In our study, subjective discomfort was relatively severe in the FS-LASIK group compared with that in the SMILE group during the very early phase after surgery. It is necessary to further verify whether this is related to the more severe dry eye symptoms in the FS-LASIK group in the late postoperative period. Although there were fewer subjective discomfort reports in the SMILE group than in the FS-LASIK group, visual acuity recovery and optical quality was poor at 2 h and 4 h after SMILE surgery compared with FS-LASIK. This delay might be associated with the formation of interface haze in the early postoperative period.

Despite being the first report on clinical parameters in the very early phase after SMILE or FS-LASIK, the study has some limitations. The first is that the sample size was small and not adequate to reflect the visual recovery of patients in general. Additionally, the clinical parameters reflected by vision tests may be affected by a variety of factors, such as ametropia and physical and mental conditions. A larger cohort of subjects should be evaluated to assess the parameters of vision recovery to a greater degree. Another limitation is that the details of optical quality, which are as important as visual quantity, were not assessed. More detailed analyses should be performed to compare the efficacy of the two surgical procedures.

## Conclusions

In conclusion, our prospective study supports that both SMILE and FS-LASIK procedures are effective in the treatment of myopia after evaluations in terms of MTF cutoff, OSI and SR. Visual and optical quality outcomes of FS-LASIK for myopia were better than those of SMILE in the very early phase after surgery, which is attributed to the formation of interface haze.

## Abbreviations

CCT: central corneal thickness
CDVA: corrected distance visual acuity
FS-LASIK: femtosecond laser-assisted in situ keratomileusis
IOP: intraocular pressure, VA:visual acuity
MTF: Cutoff for Modulation Transfer Function
OQAS: Optical Quality Analysis System
OSI: Objective Scattering Index
SE: spherical equivalent
SMILE: Small incision lenticule extraction
SR: Strehl ratio
